# Ethnobotanical investigations among tribes in Madurai District of Tamil Nadu (India)

**DOI:** 10.1186/1746-4269-2-25

**Published:** 2006-05-11

**Authors:** S Ignacimuthu, M Ayyanar, Sankara Sivaraman K

**Affiliations:** 1 Entomology Research Institute, Loyola College, Chennai, India; 2 Centre for Research & Post Graduate Studies in Botany, AyyaNadar JanakiAmmal College, Sivakasi, India

## Abstract

**Background:**

An ethnobotanical survey was carried out to collect information on the use of medicinal plants in Southern Western Ghats of India (Madurai district, Tamil Nadu). Information presented in this paper was gathered from the *paliyar *tribes using an integrated approach of botanical collections, group discussions and interviews with questionnaires in the years 1998 – 1999. The informants interviewed were 12 among whom 4 were tribal practitioners.

**Results:**

A total of 60 ethnomedicinal plant species distributed in 32 families are documented in this study. The medicinal plants used by *paliyars *are listed with Latin name, family, local name, parts used, mode of preparation and medicinal uses. Generally, fresh part of the plant was used for the preparation of medicine.

**Conclusion:**

We observed that the documented ethnomedicinal plants were mostly used to cure skin diseases, poison bites, stomachache and nervous disorders. The results of this study showed that these tribal people still depend on medicinal plants in Madurai district forest areas.

## Background

Globally, about 85% of the traditional medicines used for primary healthcare are derived from plants [1]. Traditional medicine and ethnobotanical information play an important role in scientific research, particularly when the literature and field work data have been properly evaluated [2]. India is one of the twelve mega-biodiversity countries of the World having rich vegetation with a wide variety of plants with medicinal value. In many countries, scientific investigations of medicinal plants have been initiated because of their contribution to healthcare. Herbal medicines have good values in treating many diseases including infectious diseases, hypertension, etc. That they can save lives of many, particularly in the developing countries, is undisputable [3].

India possesses a total of 427 tribal communities [4] and over 275 papers have been published on specific ethnic groups [5]. Interest in traditional medicine in India has continuously been increasing; recently, various ethnobotanical studies have been reported to explore the knowledge from the various tribals of Tamil Nadu [6-13].

Even today many local and indigenous communities in the Asian countries meet their basic needs from the products they manufacture and sell based on their traditional knowledge. Herbal drugs obtained from plants are believed to be much safer; this has been proved in the treatment of various ailments [14]. Rural communities, in particular paliyar tribes, depend on plant resources mainly for herbal medicines, food, forage, construction of dwellings, making household implements, sleeping mats, and for fire and shade. Rural people not only depend on wild plants as sources of food, medicine, fodder and fuel, but have also developed methods of resource management, which may be fundamental to the conservation of some of the world's important habitats [15].

The objective of this study was to assess the richness of ethnomedicinal plant species used by the paliyar tribes in Madurai district forest areas and the traditional medical practices of the people. Similar ethnobotanical studies have been reported in several parts of India to protect the traditional knowledge from disappearing [5,12,16-19]. Documenting the indigenous knowledge through ethnobotanical studies is important for the conservation of biological resources as well as their sustainable utilization.

## Setting and the people

### *Paliyar *tribals

The indigenous people of the study area are called *Paliyar/Paliyan*. They are found in the hilly regions of Madurai, Dindigul, Theni, Tirunelveli and Virudhunagar districts. It is believed that *Paliyars *are indigenous people of Palani hills (Situated near to Kodaikanal – a famous tourist place). In the Palani hills they are found at an altitude of upto 2200 m. Generally *paliyars *are illiterate and they speak Tamil (Mother tongue of Tamil Nadu). *Paliyars*, when compared to various tribal communities in Tamil Nadu constitute relatively a small group [20].

Physically they are similar to the *Semong *of Malaya and other Indian tribal communities [21,22]. Historically, these tribal communities have survived on their traditional knowledge base. Traditional medicines are the primary healthcare resources for the *paliyar *tribes to protect their health. Tribal practitioners are the curators of the tribal society and they have a good knowledge of medicinal plants, diseases and treatment using plants.

### Type of *paliyars*

*Paliyars *can be grouped into three categories based on their life styles, namely, nomadic, semi nomadic and settled. Nomadic *paliyars *don't build houses; they live temporarily in rock caves called 'pudai'. Semi nomadic *paliyars *build temporary houses and confine themselves to small territories. Most of their huts are dark with no window or any other opening to admit air. Settled *Paliyars *are more or less urbanized and live as agricultural laborers. Importance of traditional and folk medicine in the treatment of various human ailments is well recognized amongst these people.

## Methods

### Description of the study area

**Figure 1:**
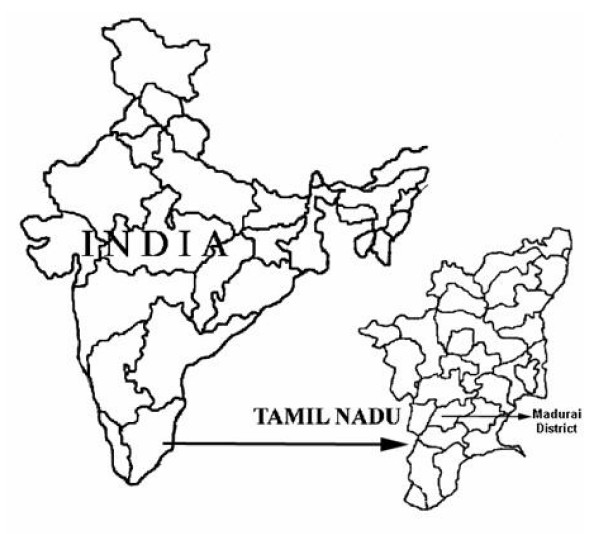
Location of the area studied in Tamil Nadu, India.

The Western Ghats, a chain of mountains in the western peninsular India extending from Tapti river valley in Gujarat to Kanyakumari in Tamil Nadu, is about 1600 km long in North-South direction. The study area concentrates in and around the Madurai district forest areas (Figure. 1) located in Tamil Nadu, South India. The area of investigation approximately lies between 85°0' to 89°0' longitude and 28°0' 37° to 0° latitude. Every village has several paliyar hamlets. Their hamlets are found in different elevations from 300 m to 2,200 MSL. There are a number of hill ranges in the study area. Temperature ranges from 12° to 25° during March – April in high hill ranges and averages between 20° during December and 38° during April – May.

### Ethnobotanical survey

The fieldwork was conducted in several villages around Madurai district forest areas during April 1998 to November 1999 as part of a study of Ethnobotanical Wealth of *Paliyar *Tribals in Tamil Nadu [20]. More than 500 families and nearly 3000 members of *paliyars *are found in the study area. During the stay, their daily activities were closely observed and interpersonal contacts were established by participating in several of their social and religious ceremonies such as marriages, rituals and curing sessions. There were 12 informants (9 males and 3 females) between the ages of 32 to 78 in the study area. Among them 5 were farmers, 3 were housewives and 4 regular tribal practitioners.

### Interviews with tribal practitioners

Ethnobotanical data were collected according to the methodology suggested by Jain [23]. The ethnobotanical data (local name, mode of preparation, medicinal uses) were collected through questionnaire, interviews and discussions among the tribal practitioners in their local language. Our questionnaire allowed descriptive responses on the plant prescribed, such as part of the plant used, medicinal uses, detailed information about mode of preparation (i.e., decoction, paste, powder and juice), form of usage either fresh or dried and mixtures of other plants used as ingredients. The Flora of Presidency of Madras [24] and The Flora of Tamil Nadu Carnatic [25] were used to ascertain the nomenclature. The voucher specimens in duplicate were deposited in the herbarium of Entomology Research Institute, Loyola College, Chennai (India).

## Results and discussion

In Table 1, data obtained from the field survey are presented. In this study 60 plant species belonging to 32 families distributed in 53 genera have been recorded. These contribute to 81 remedies. Many species of the family Acanthaceae and Asclepiadaceae are frequently used (21 remedies from 12 species); the Euphorbiaceae and Solanaceae contribute to 9 remedies from 8 species. The informations collected from this study are in agreement with the previous reports [5-12], [16-19].

**Table 1:** Ethnomedicinal plant species, plant parts used and ailments cured by the Paliyar tribes in Madurai District of Tamil Nadu, India

Therapeutic indication and associated plants	Local Name	Family	Parts used and Ethnomedicinal preparation
**Asthma**			
*Solanum trilobatum *L.	Thoodhuvalai	Solanaceae	Juice of leaves is taken orally for seven days.
*Adhatoda zeylanica *Medicus.	Adathodai	Acanthaceae	Leaf paste is taken orally.
**Cold**			
*Adhatoda zeylanica *Medicus.	Adathodai	Acanthaceae	Leaf powder is mixed with water and taken orally in the morning.
*Boswellia serrata *Roxb. Ex Colebr.	Kungiliyam	Burseraceae	Powdered resin is sprayed on burning charcoal & the smoke is inhaled.
*Plectranthus coleoides *Benth.	Omavalli chedi	Lamiaceae	Juice of leaves is taken internally.
*Solanum trilobatum *L.	Thoodhuvalai	Solanaceae	Juice of leaves is taken orally for seven days early morning until cure.
*Terminalia chebula *Retz.	Kadukkai maram	Combretaceae	Powdered fruit is mixed with water or cow's or goat's milk and taken internally.
*Vitex negundo *L.	Notchi	Verbenaceae	Fresh leaves are boiled with water and the vapour is inhaled twice a day.
**Cough**			
*Adhatoda zeylanica *Medicus.	Adathodai	Acanthaceae	Leaf powder is mixed with water and taken orally in the morning.
*Terminalia chebula *Retz.	Kadukkai maram	Combretaceae	Powdered fruit is mixed with the water or cow's or goat's milk and taken internally.
*Vitex negundo *L.	Notchi	Verbenaceae	Fresh leaves are boiled with water and the vapour is inhaled twice a day.
**Diabetes**			
*Andrographis lineata *Wallich ex Nees.	Siriyanangai	Acanthaceae	Leaf powder is mixed with cow's or goat's milk and taken orally.
*Costus speciosus *(J. Koen.) Smith.	Koshtam	Zingiberaceae	Powdered leaves are taken internally with cow's milk.
*Gymnema sylvestre *(Retz.) R. Br. ex Roem. & Schult.	Sirukurinjan	Asclepiadaceae	Powdered leaves are mixed with cow's milk and boiled rice, kept over night and taken internally twice a day.
**Diarrhoea**			
*Cipadessa baccifera *(Roth.) Miq.	Pulippan chedi	Meliaceae	Paste of leaves is mixed with the cup of water or milk and taken orally.
**Dysentery**			
*Acalypha fruticosa *Forsskal.	Chinni chedi	Euphorbiaceae	Decoction of leaves taken orally.
**Eye infections**			
*Alangium salvifolium *(L.f.) Wangerin.	Alinji	Alangiaceae	One or two drops of fruit juice is poured in the eyes.
**Fever**			
*Adhatoda zeylanica *Medicus.	Adathodai	Acanthaceae	Leaf decoction is taken internally twice a day until cure.
*Hemidesmus indicus *H.f.	Nannari	Asclepiadaceae	Decoction of whole plant is taken internally.
*Terminalia chebula *Retz.	Kadukkai maram	Combretaceae	Powdered fruit is mixed with the water or cow's or goat's milk and taken internally.
*Vitex negundo *L.	Notchi	Verbenaceae	Fresh leaves are boiled with water and the vapour is inhaled twice a day.
**Headache**			
*Ceropegia candelabrum *L.	Perun kodi	Asclepiadaceae	Paste of leaves is applied on forehead.
*Pergularia daemia *(Fors.) Chiov.	Veli parutthi	Asclepiadaceae	Fresh leaves are boiled with water and the vapour is inhaled.
*Vitex negundo *L.	Notchi	Verbenaceae	Fresh leaves are boiled with water and the vapour is inhaled twice a day.
**Heel cracks**			
*Asparagus racemosus *Willd.	Thanneer vittan kilangu	Liliaceae	Paste of tender and mature leaves is applied topically on the heels before going to bed.
*Drymaria cordata *(L.) Roem. & Schult.	Kodi charai	Caryophyllaceae	Paste of leaves is applied over the heels before going to bed regularly till cure.
*Rubia cordifolia *L.	Kalutharupan chedi	Rubiaceae	Root paste is applied topically on heel before going to bed.
**Jaundice**			
*Centella asiatica *(L.) Urban.	Vallarai	Umbelliferae	Juice of leaf is mixed with equal amount of goat's milk and taken orally for seven days.
**Menorrhagia**			
*Hemidesmus indicus *H.f.	Nannari	Asclepiadaceae	Paste of root is mixed with water or cow's milk and taken internally twice a day.
**Nervous disorders**			
*Bischofia javanica *Blume.	Romaviruksha pattai	Bischofiaceae	Paste of stem bark is applied externally on the affected parts.
*Blepharis maderaspatensis *(L.) Roth.	Vettukaaya pachilai	Acanthaceae	Leaf paste is mixed with the powdered black gram, crushed onion and white yolk of one egg and the mixture is applied topically over the fractured bones.
*Euphorbia antiquorum *L.	Sathura kalli	Euphorbiaceae	Stem latex is applied topically on skin to get relief from body pain.
*Gymnema sylvestre *(Retz.) R. Br. ex Roem. & Schult.	Sirukurinjan	Asclepiadaceae	Paste of leaves is applied externally.
*Phlebophyllus kunthianum *Nees.	Kurinji chedi	Acanthaceae	Fresh leaves and bark are heated with gingelly oil and applied externally on affected part of the body.
**Piles**			
*Gmelina arborea *Roxb.	Perungilai/Kumilamaram	Verbenaceae	Juice of root bark is taken internally.
**Pimples**			
*Acalypha paniculata *Miq.	Paruva thazhai	Euphorbiaceae	Leaf paste is applied over pimples regularly once a day until cure.
**Poison bites**			
*Andrographis lineata *Wallich ex Nees.	Siriyanangai	Acanthaceae	Paste of leaves is applied externally on bitten site of scorpion and snake.
*Andrographis paniculata *(Burm.f.) Wall. ex Nees.	Periyanangai or Nilavembu	Acanthaceae	Paste of leaves is applied externally on bitten site of scorpion sting and snakebites.
*Tylophora indica *(Burm. f.) Merr.	Nangilai	Asclepiadaceae	Paste of leaf and root is mixed with equal amount of root paste of *Rauvolfia serpentina *and applied externally on the spot of snakebite. Leaf juice alone is also taken internally to cure snakebite.
**Skin diseases**			
*Acalypha fruticosa *Forsskal.	Chinni chedi	Euphorbiaceae	Leaf and root paste is applied topically on the affected places.
*Anisochilus carnosus *(L.f.) Wallich.	Saetthupun thazhai	Lamiaceae	Paste of leaves is applied over the affected places.
*Balanophora fungosa *Fors and Fors. var. indica.	Vaer chedi	Balanophoraceae	Paste of the whole plant is applied over the infected part of the skin.
*Clematis gouriana *Roxb. Ex. DC.	Attumeesai chedi	Ranunculaceae	Paste of leaves is applied topically on affected part of the skin.
*Excoecaria crenulata *L.	Vellai thillai	Euphorbiaceae	Paste of the stem is applied on the affected part of the skin.
*Lobelia heyneana *Roem. & Schult.	Upperi chedi	Lobeliaceae	Leaves and flowers are mixed with water and the paste is applied on skin till cure.
*Mahonia leschenaultii *(Wight & Arn.) Tak. ex Gamble	Mullu kadambu	Berberidaceae	Powdered stem bark is boiled with gingelly oil and applied over the body before bath.
**Stomachache**			
*Acalypha paniculata *Miq.	Paruva thazhai	Euphorbiaceae	Juice of leaves is taken orally.
*Dioscorea oppositifolia *L. var. tomentosa.	Nurulai/Valli kilangu	Dioscoreaceae	Paste of rhizome is taken internally.
*Elatteria cardamomum *(L.) Maton.	Yelakkai	Zingiberaceae	Dried fruits are taken internally with food.
*Hemidesmus indicus *H.f.	Nannari	Asclepiadaceae	Fresh leaves are taken internally.
*Plumbago zeylanica *L.	Chitthira moolam	Plumbaginaceae	Powdered root is mixed with goat's milk and taken internally.
*Solanum nigrum *L.	Mana thakkali	Solanaceae	Fresh leaves are cooked with onion bulbs and cumin seeds and taken along with food regularly.
*Terminalia chebula *Retz.	Kadukkai maram	Combretaceae	Powdered fruit is mixed with water or cow's or goat's milk and taken internally.
*Toddalia asiatica *(L.) Lam.	Kindu mullu	Rutaceae	Decoction of leaves is given internally.
**Throat infection**			
*Acorus calamus *L.	Vasambu	Araceae	Dried rhizome is rubbed on stone with water and one or two drops of watery paste are given orally to the children for clarity of speech. Increased dosage will affect speech.
*Piper nigrum *L.	Milagu	Piperaceae	The dried seeds are taken orally.
**To increase lactation**			
*Alstonia scholaris *(L.) R.Br.	Paalooram pattai	Apocynaceae	Powdered stem is mixed with water and given orally to the mother.
**To increase resistance power**			
*Alpinia calcarata *Rosc.	Arathi poo	Zingiberaceae	Dried rhizome is mixed with water and two drops of juice are given orally to children.
**To induce fertility**			
*Carmona retusa *(Vahl) Masam.	Kurangu vetthilai	Boraginaceae	Juice of leaves is taken internally for three to four months.
**Toothache**			
*Solanum erianthum *D.Don	Malai sundai	Solanaceae	The ripened or unripened fruits are boiled with water and the vapour is inhaled once or twice a week through mouth.
*Solanum surattrense *Burm. f.	Kandankathiri	Solanaceae	Fresh or dried fruits are kept in fire and the smoke is inhaled with mouth.
*Toddalia asiatica *(L.) Lam.	Kindu mullu	Rutaceae	Powder of root and stem bark is used as tooth powder.
**To reduce delivery time pain**			
*Plectranthus coleoides *Benth.	Mudupattan or Omavalli chedi	Lamiaceae	Leaf juice is taken internally by pregnant women.
*Pterolobium hexapetalum *(Roth.) Sant. & Wagh.	Kari indu	Caesalpiniaceae	Decoction of leaves is taken internally by pregnant women.
**To stimulate appetite**			
*Asystasia gangetica *(L.) T. Anderson.	Valukai keerai	Acanthaceae	Fresh leaves are cooked with cumin seeds and onion bulbs and taken orally with food.
**To stimulate Hair growth**			
*Bischofia javanica *Blume.	Romaviruksha pattai	Bischofiaceae	Stem bark is mixed with coconut oil and applied over head.
*Plectranthus coleoides *Benth.	Omavalli chedi	Lamiaceae	Juice of leaves is boiled with coconut oil and applied on head.
*Sida acuta *Burm. f.	Pilla valatthi chedi.	Malvaceae	Paste of leaves is mixed with coconut oil and applied on head regularly for killing dandruffs and also for strengthening hair.
**To stupefy fish**			
*Catunaregum spinosa *(Thun.) Tiruvengadam	Karangai maram	Rubiaceae	Crushed unripe fruits are used.
*Sapindus emarginata *Vahl.	Poondi kottai	Sapindaceae	Unripe fruits are crushed and thrown on the running and stagnant water.
**Menstrual disorders**			
*Andrographis paniculata *(Burm. f.) Wall. ex Nees.	Periyanangai or Nilavembu	Acanthaceae	Leaf juice is taken orally during menstruation to prevent excessive bleeding.
**Wounds**			
*Acacia caesia *(L.) Willd.	Nanjupattai	Mimosaceae	Bark is ground with water and applied topically over the affected part.
*Acacia leucophloea *(Roxb.) Willd.	Sarayapattai maram	Mimosaceae	Paste of fresh bark is applied topically on cuttings until cure.
*Anisomeles malabarica *(L.) R. Br. Ex. Sims.	Paei miratti	Lamiaceae	Paste of stem is mixed with coconut oil and applied over the affected places.
*Blepharis maderaspatensis *(L.) Roth.	Vettukaaya pachilai	Acanthaceae	Paste of leaves is mixed with limejuice and applied on cuts.
*Clausena dentata *(Willd.) Roem.	Anai thazhai	Rutaceae	Paste of leaves is applied over the affected parts.
*Cryptolepis buchananii *Roem & Schul.	Paalkodi/Karunkodi	Asclepiadaceae	Stem latex (5 – 10 drops) is applied on the affected places.
*Plectranthus coleoides *Benth.	Omavalli chedi	Lamiaceae	Paste of leaves is applied over wounds till cure.
*Solanum nigrum *L.	Mana thakkali	Solanaceae	Fresh leaf paste is applied externally on cuts.
*Sonchus oleraceus *L.	Kaalaadi pachilai	Asteraceae	Leaves are ground with the equal amount of leaves of *Smilax zeylanica *and applied externally on the cuts once a day till cure.

As seen in Table 1, common health ailments in the study area were skin problems such as wounds, cuts, burns and skin diseases and the largest number of the remedies (16 remedies from 16 species) were used to treat these ailments. Kani tribals in Tirunelveli hills of Tamil Nadu were using 14 plants for the treatment of skin problems [13]; 52 herbal preparations from 31 plants were used for skin diseases by tribals of Uttar Karnataka district, a nearest state of Tamil Nadu [18] and people of Eastern Cape Province, South Africa used 38 plant species for the treatment of wounds [27].

On the other hand, 14 remedies were used to alleviate problems of the respiratory system such as cold, cough, asthma and fever. Among the plants surveyed, *Adhatoda zeylanica *and *Vitex negundo *(4 remedies) are used frequently for the preparation of medicines for the treatment of respiratory problems. Respiratory problems are the most encountered illness and there may be hardly any person who has not suffered from respiratory problems in his lifetime [28]. 8 remedies were used against gastrointestinal problems such as stomachache, abdominal pain and ulcer. A previous study (29) has reported the use of 21 medicinal plants from 20 families to treat gastro-intestinal complaints in the same community. 5 remedies were used against inflammatory diseases such as rheumatism, fractured bones and joint pains.

Leaves of *Acacia caesia*, *Asystasia gangetica*, *Oxalis corniculata*, rhizomes of *Costus speciosus*, *Dioscorea opositifolia*, fruits of *Gmelina arborea*, and seeds of *Piper nigrum *are used as edible plants by the local inhabitants of the study area. The tribal people mostly eat vegetables of leafy varieties, which grow as wild weeds.

There are two types of tribal healers found in the study area namely herbalists and ritualists. Herbalists treat patients only by using plant resources. They diagnose diseases based on the symptoms told by the patients as well as based on their personal experience in treating human ailments. Ritualists believe that specific spirit causes ailments. The whole healing ceremony takes about a day. The preparation of medicines and treatment of diseases connected with the tribal healthcare are accompanied by elaborate rituals and music as previously observed in the case of *Mikirs *of India [30].

Paliyar tribal practitioners use specific plant parts and specific dosages for the treatment of ailments. The plant products are consumed raw or in the form of a decoction, as infusion for oral treatment and as burnt product, ointments or raw paste when applied externally. The parts of the plant most used for medicinal purposes are leaves, root, stem, fruits, the complete aerial parts, the whole plant, barks (root and stem) and flowers (including the flowering heads) in decreasing order. Internal uses (used in 36 of the cases) are predominating over external (used in 30 cases) uses. Juice (almost mix with water and goat's or cow's milk) and paste are the main methods of preparation, either for oral or for external administration. For topical use, the most important methods used are direct application of the paste or ointment (with oil).

Often, people use more than one plant either separately or mixed together. They mix several plants as ingredients to cure diseases immediately. Generally, fresh part of the plant is used for the preparation of medicine. When fresh plant parts are not available, dried parts are also used. Majority of medicinal plants are used as simple drugs and some plants are used with some other plant parts.

Paliyar tribes use some plants to prepare agricultural implements. For example *Terminalia bellirica *and *Haldenia cordifolia *are used to prepare ploughs; they prepare musical instruments from the wood of *Gmelina arborea*. They crush the fruits of *Solanum erianthum *and apply topically on their legs, while entering into forest to protect from leech-bite. From this account it is clear that the *paliyar *tribals like other ancient tribals [31] possess the ability to discern the character of various plants and their beneficial properties. It is interesting to note that such a way of life, particularly with respect to healthcare practices has hardly undergone any change even in the present day.

## Conclusion

This study shows that knowledge and usage of herbal medicine for the treatment of various ailments among paliyar tribes is still a major part of their life and culture. They use forest plants, weeds, fruit plants, vegetables, spices, ornamental plants, ferns and many others as traditional medicine. Although many of these species are known as medicinal plants, others are mainly used for nonmedicinal purposes such as preparing agricultural implements. *Adhatoda zeylanica*, *Vitex negundo*, *Plectranthus coleoides*, and *Piper nigrum *are the leading species used as remedies against a variety of complaints.

The data collected show that majority of the remedies are taken orally. Most of the reported preparations are drawn from a single plant; mixtures are used rarely. In other parts of the country, the use of mixtures of plant species in treating a particular ailment is fairly common [6-13]. Generally, the people of the study area still have a strong belief in the efficacy and success of herbal medicine. The results of the present study provide evidence that medicinal plants continue to play an important role in the healthcare system of this tribal community.
